# RETRACTED: Thiyagarajan, J.S. Non-Destructive Testing Mechanism for Pre-Stressed Steel Wire Using Acoustic Emission Monitoring. *Materials* 2020, *13,* 5029

**DOI:** 10.3390/ma18122812

**Published:** 2025-06-16

**Authors:** Jothi Saravanan Thiyagarajan

**Affiliations:** School of Infrastructure, Indian Institute of Technology Bhubaneswar, Argul-Jatni Rd, Kansapada, Odisha 752050, India; tjs@iitbbs.ac.in

The journal retracts the article “Non-Destructive Testing Mechanism for Pre-Stressed Steel Wire Using Acoustic Emission Monitoring” [1], cited above.

Following publication, concerns were brought to the attention of the publisher regarding an overlap between this paper [1] and an early thesis [2], published in another language by a different author.

Adhering to our complaints procedure, an investigation was conducted by the Editorial Office and Editorial Board that confirmed a significant overlap without appropriate acknowledgement or citation. As a result, the *Materials* Editorial Office and Editorial Board have decided to retract [1] this paper as per MDPI’s retraction policy (https://www.mdpi.com/ethics#_bookmark30, accessed on 1 November 2023).

This retraction was approved by the Editor-in-Chief of the journal *Materials*.

The author did not provide a comment on this decision.
